# Unveiling the effect of dietary essential oils supplementation in *Sparus aurata* gills and its efficiency against the infestation by *Sparicotyle chrysophrii*

**DOI:** 10.1038/s41598-020-74625-5

**Published:** 2020-10-20

**Authors:** Joana P. Firmino, Eva Vallejos-Vidal, Carmen Sarasquete, Juan B. Ortiz-Delgado, Joan Carles Balasch, Lluis Tort, Alicia Estevez, Felipe E. Reyes-López, Enric Gisbert

**Affiliations:** 1 IRTA, Centre de Sant Carles de la Ràpita (IRTA-SCR), Aquaculture Program, Crta. Poble Nou km 5.5, 43540 Sant Carles de la Ràpita, Spain; 2 TECNOVIT-FARMFAES, S.L. Pol. Ind. Les Sorts, parc. 10, 43365 Alforja, Spain; 3 PhD Program in Aquaculture, Universitat Autònoma de Barcelona, 08193 Bellaterra, Spain; 4 Centro de Biotecnología Acuícola, Departamento de Biología, Facultad de Química y Biología, Universidad de Santiago de Chile, Santiago, Chile; 5 Instituto de Ciencias Marinas de Andalucía (ICMAN-CSIC), Universidad de Cádiz, Campus Universitario Río San Pedro, Puerto Real, Cádiz, Spain; 6 Department of Cell Biology, Physiology and Immunology, Universitat Autònoma de Barcelona, 08193 Bellaterra, Spain

**Keywords:** Immunology, Infection, Innate immunity, Molecular biology, Transcriptomics, Pathogens, Gene expression analysis, Microarray analysis

## Abstract

A microencapsulated feed additive composed by garlic, carvacrol and thymol essential oils (EOs) was evaluated regarding its protective effect in gills parasitized by *Sparicotyle chrysophrii* in *Sparus aurata*. A nutritional trial (65 days) followed by a cohabitation challenge with parasitized fish (39 days) were performed. Transcriptomic analysis by microarrays of gills of fish fed the EOs diet showed an up-regulation of genes related to biogenesis, vesicular transport and exocytosis, leukocyte-mediated immunity, oxidation–reduction and overall metabolism processes. The functional network obtained indicates a tissue-specific pro-inflammatory immune response arbitrated by degranulating acidophilic granulocytes, sustained by antioxidant and anti-inflammatory responses. The histochemical study of gills also showed an increase of carboxylate glycoproteins containing sialic acid in mucous and epithelial cells of fish fed the EOs diet, suggesting a mucosal defence mechanism through the modulation of mucin secretions. The outcomes of the in vivo challenge supported the transcriptomic results obtained from the nutritional trial, where a significant reduction of 78% in the abundance of *S. chrysophrii* total parasitation and a decrease in the prevalence of most parasitic developmental stages evaluated were observed in fish fed the EOs diet. These results suggest that the microencapsulation of garlic, carvacrol and thymol EOs could be considered an effective natural dietary strategy with antiparasitic properties against the ectoparasite *S. chrysophrii*.

## Introduction

Nutritional therapies provide an important strategy for preventing and/or treating diseases^1^. Among different options, such as the use of probiotics, prebiotics, immunostimulants and organic acids, phytogenics have gained interest as feed additives within aquafeeds^2^. Phytogenics are plant-based natural substances derived from herbs, spices or extracts similar to essential oils (EOs), which are reputed for their beneficial properties and efficacy on performance and health in animal production^3^. In aquafeeds, EOs as dietary additives have been reported to stimulate appetite, improve feed utilization and growth, and boost the innate immunity^4^.

Thymol, carvacrol, cinnamaldehyde and EOs from clove, coriander, star anise, ginger, garlic, rosemary, mint among others, have been used either individually or as blends in animal nutrition^5,6^. Among phytogenics, oregano (*Origanum vulgare*) is the most common because of its richness in carvacrol and thymol^7,8^. These compounds have a wide range of properties such as antimicrobial^9^, immunostimulant and anti-oxidative activities^10,11^, and the ability to enhance intestinal absorption^12^, to improve growth^13^ and even to reduce cumulative mortality^11^. The effectiveness of garlic (*Allium sativum*) extract as an immunostimulant, antimicrobial and antiparasitic agent has been demonstrated in several fish species^14–18^; the inclusion of garlic extract in fish diets is effective against monogenean parasites^19,20^.

Combinations of different EOs are promising strategies for functional feeds; however, evaluating their effectiveness in front of a biological challenge such as an ectoparasite infestation, as well as deciphering their mode of action is necessary *prior* to their recommendation as feed additives^21^. Under this context, the aim of this work was to evaluate the functional response of gilthead seabream, the most important farmed fish in the Mediterranean basin, to the dietary administration of a microencapsulated combination of garlic, carvacrol and thymol EOs. In teleosts, gills are one of the main mucosal barriers containing an associated-lymphoid tissue (GIALT) with innate and adaptive immune components that pathogens encounter upon first contact with the host^22,23^. Thus, we ran a nutritional trial with the assessment of gill’s transcriptomic profiling in order to describe for the first time the main metabolic and immune pathways regulated by these EOs in this lymphoid tissue, as well as the histochemical properties of mucins produced by branchial mucous cells. Moreover, the efficiency in controlling the infestation by *S. chrysophrii* was also assessed through an in vivo cohabitation challenge trial.

## Results

### Growth performance

At the end of the nutritional period or at the end of the *S. chrysophrii* challenge, no differences in SR, BW, SL, K or SGR_BW_ were found between fish fed both diets (Table 1; P > 0.05).

**Table 1:** Body weight (BW, standard length (SL), Fulton’s condition index (K), specific growth rate for body weight (SGR_BW_) (mean ± SD) and survival rate (SR) of juvenile gilthead seabream fed with the control and garlic, carvacrol and thymol essential oils (EOs) experimental diets at the beginning of the nutritional trial (day 0), at the beginning of the cohabitation trial with *S. chrysophrii* (day 65) and at the end of the study (day 104).

Diets	Nutritional trial					
	Day 0		Day 65		Day 104	
	Control	EOs	Control	EOs	Control	EOs
BW (g)	40.2 ± 4.7	40.4 ± 5.1	157.8 ± 14.2	150.8 ± 14.9	205.4 ± 23.9	195.4 ± 21.7
SL (cm)	11.8 ± 0.4	11.9 ± 0.5	17.3 ± 0.6	17.1 ± 0.6	18.3 ± 0.5	18.2 ± 0.4
K	2.3 ± 0.2	2.4 ± 0.1	3.1 ± 0.1	3.0 ± 0.1	3.2 ± 0.2	3.1 ± 0.2
SGR_BW_ (%)	–	–	2.12 ± 0.07	2.03 ± 0.01	1.60 ± 0.18	1.52 ± 0.17
SR (%)			92	96	100	100
			Cohabitation trial			

No significant differences were observed between dietary groups (P > 0.05).

### Gill’s transcriptomic profile

**Figure 1:**
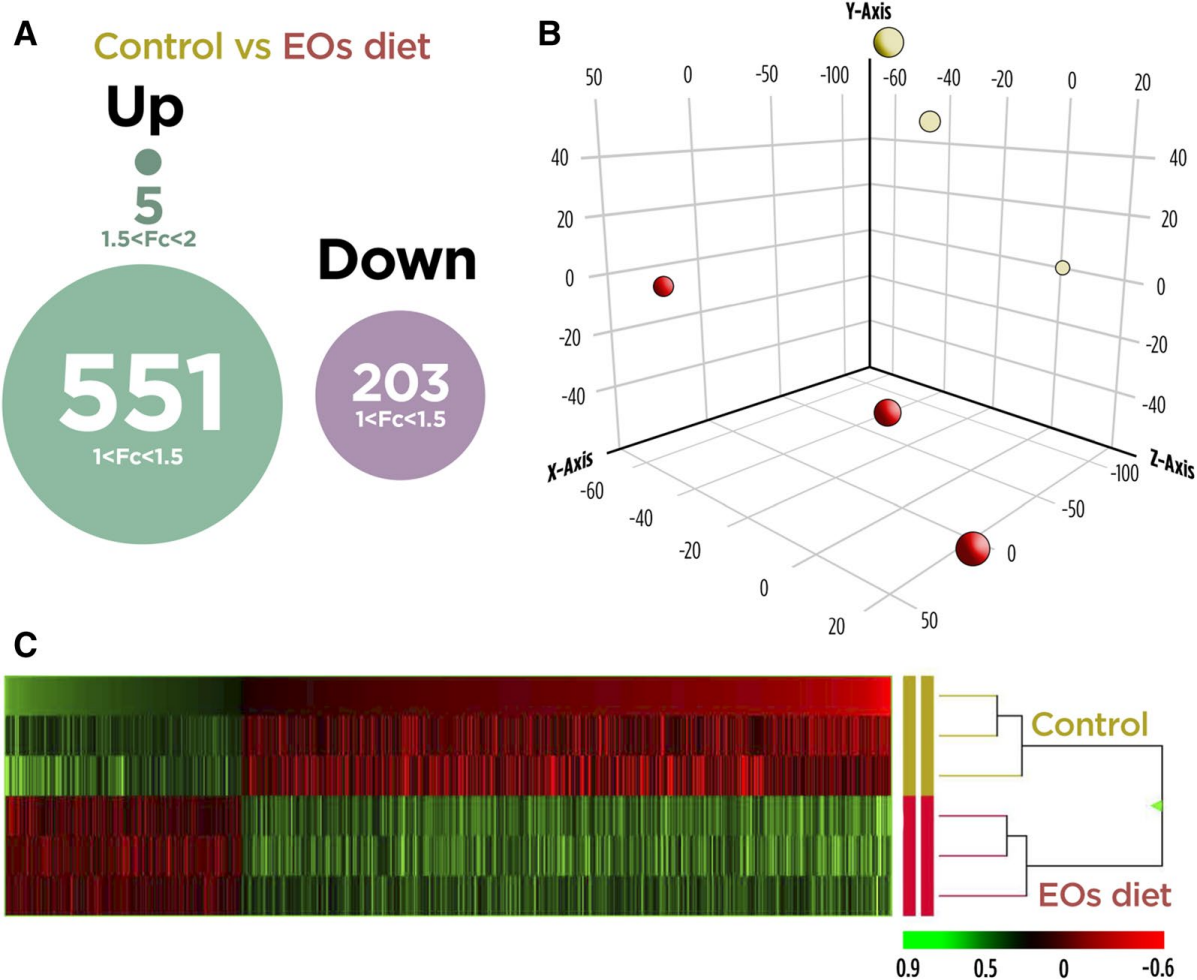
(**A**) Differential expression analysis of the gilthead seabream gill’s transcriptomic response to the garlic, carvacrol and thymol essential oils (EOs) supplemented diet. Comparing both groups, 759 DEGs (P < 0.05) were found. From these, 556 genes were up-regulated: 551 mainly concentrated in the 1.0- to 1.5-fold change (FC) interval; and 5 DEGs were grouped in the 1.5 ≤ FC ≤ 2.0 interval. Additionally, 203 genes were down-regulated (P < 0.05) and grouped in the range − 1.5 ≤ FC ≤ − 1.0. (**B**) Principal component analysis (PCA) of the DEGs of gilthead seabream gill’s response to the control diet and EOs supplemented diet. (**C**) Hierarchical clustering of the gilthead seabream gill’s transcriptomic response for the control diet and EOs supplemented diet, based in similitude patterns of the differentially expressed genes (DEGs) detected from three sample pools per dietary group. Data of the six microarrays are depicted, one for each represented pool. Both increased and decreased gene expression pattern is shown in green and red, respectively. All transcripts represented are statistically significant (P < 0.05).

A microarray-based transcriptomic analysis was conducted to determine the modulatory effect of dietary EOs upon the gill’s transcriptome in healthy fish. In total, 759 DEGs (P < 0.05; Supplementary Table 1) were found in gills comparing both groups. From these, 556 genes were up-regulated with 551 mainly concentrated in the 1.0- to 1.5-fold change (FC) interval. The other 5 DEGs were grouped in the 1.5 ≤ FC ≤ 2.0 interval. In contrast, 203 genes were down-regulated (P < 0.05) and grouped in the range − 1.5 ≤ FC ≤ − 1.0 (Fig. 1). These results indicated that genes were mostly up-regulated in fish fed dietary EOs and their modulation was moderated in terms of fold-change intensity.

**Figure 2:**
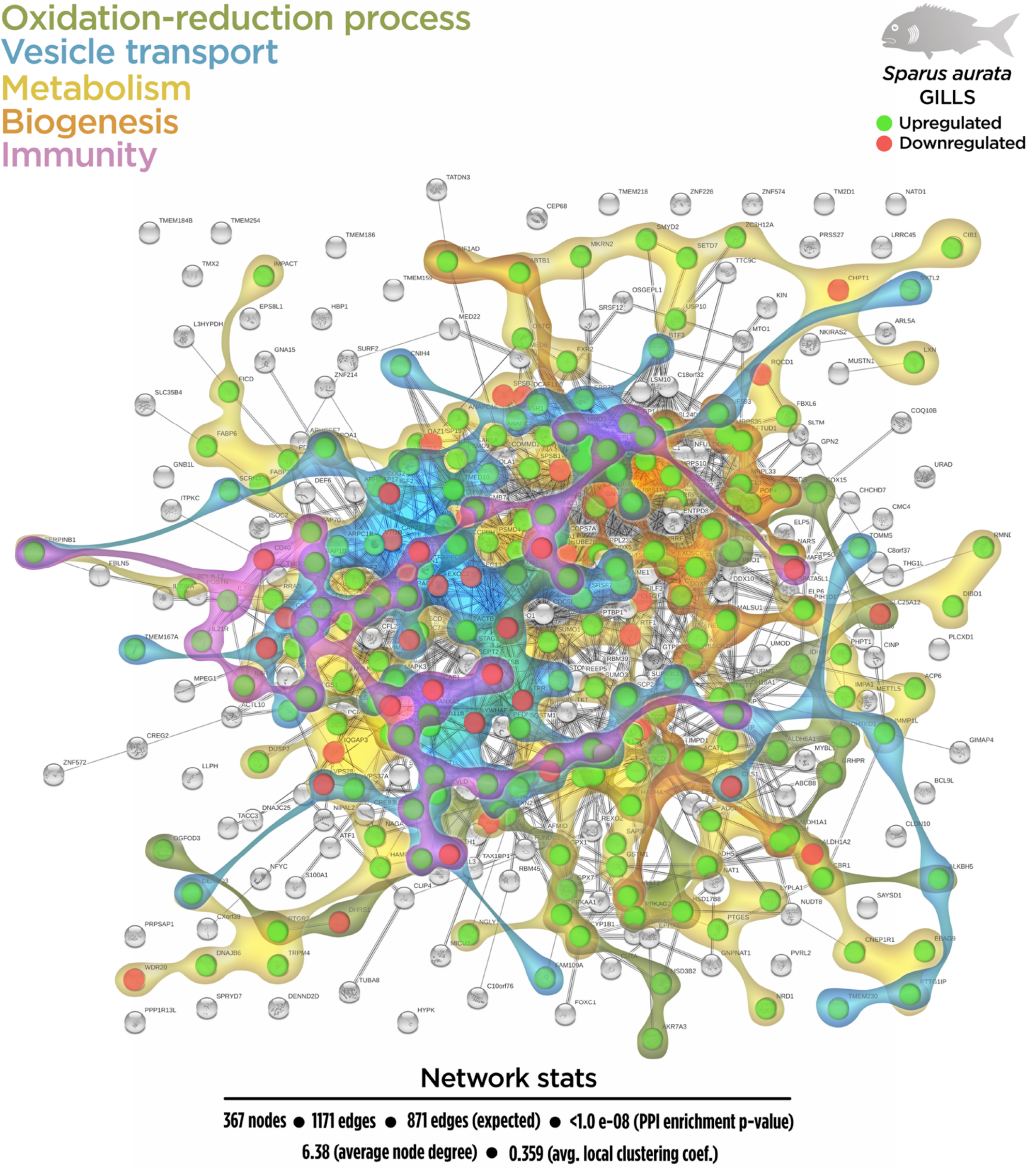
Transcripteractome of the differentially expressed genes (DEGs) in the gills of juvenile gilthead seabream fed the garlic, carvacrol and thymol essential oils (EOs) supplemented diet. Five representative processes identified from the functional enrichment analysis—biogenesis, metabolism, vesicle-mediated transport, immunity and oxidation–reduction—are highlighted distinctly in coloured amoeboid clusters in the overall Protein–Protein Interactions Network (PPI) for the DEGs (see also Supplementary Table 2). Green nodes represent up-regulated genes and red nodes represent down-regulated genes. Graphic keys including colours and network stats are indicated in the graphical figure legend.

Regarding the total DEGs, 367 nodes generated a functional network in the transcripteractome, resulting in 1171 interactions/edges (average node degree: 6.38; average local clustering coefficient: 0.359; PPI enrichment P < 1.0 e^−16^). The remaining 392 DGEs were annotated as unknown genes. From the enrichment analysis, five main representative processes (biogenesis, vesicle-mediated transport, immunity, oxidation–reduction, and metabolism; Fig. 2) were identified in the transcripteractome (Supplementary Table 2).

**Figure 3:**
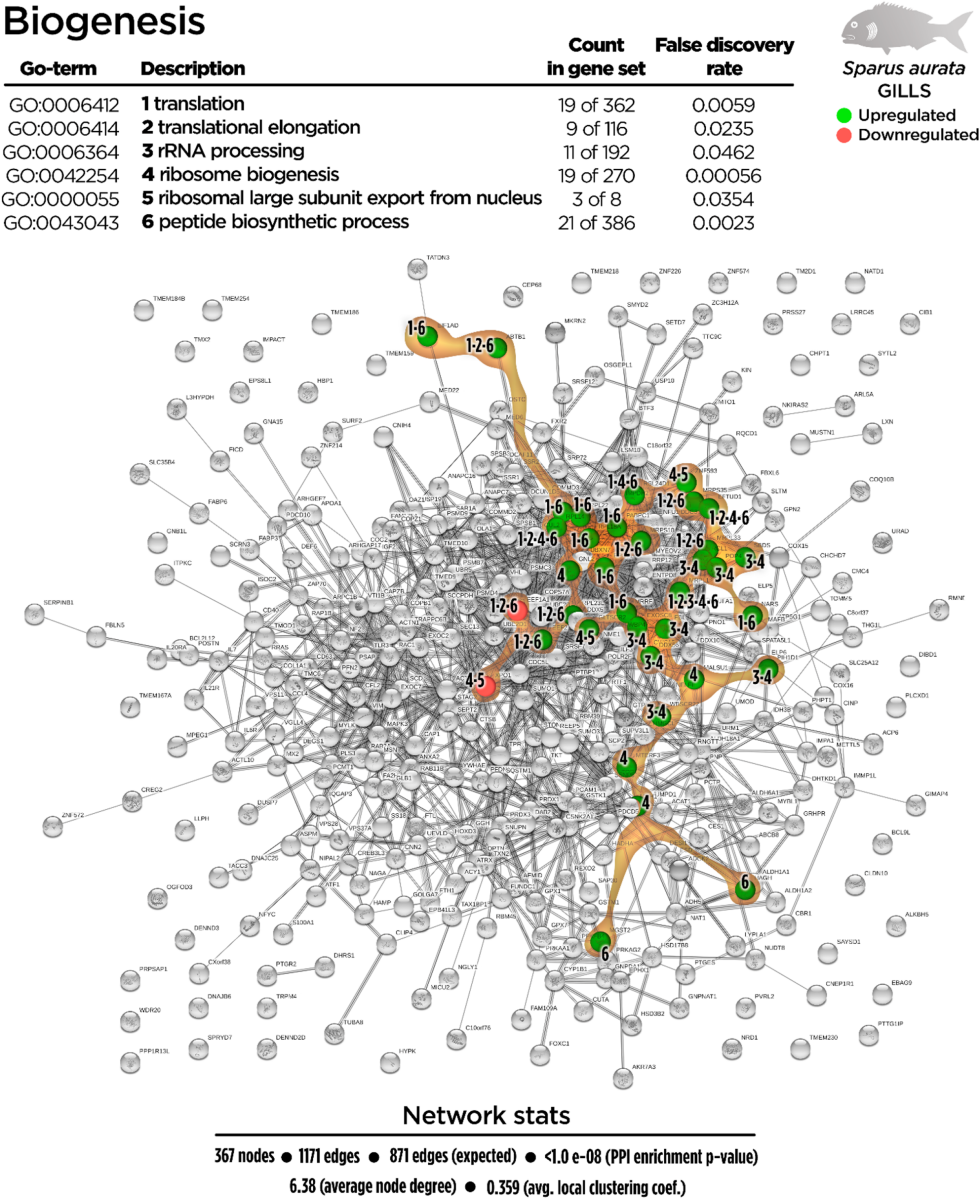
Biogenesis-related Protein–Protein Interactions Network (PPI) network for the differentially expressed genes (DEGs) in the gills of juvenile gilthead seabream fed the garlic, carvacrol and thymol essential oils (EOs) supplemented diet (see also Supplementary Table 2). Nodes numbers (1–6) indicate the biological processes for each DEG represented. Gene Ontology (GO) definitions, count of DEGs within each biological processes and respective false discovery rate are described in the graphical figure legend. Green nodes represent up-regulated genes and red nodes represent down-regulated genes. Graphic keys and network stats are indicated in the graphical figure legend.

The biological processes associated to biogenesis in the gills were favoured by dietary EOs (34 up-regulated genes; 2 down-regulated genes) (Fig. 3). Several biological processes were identified within the biogenesis process context, namely “translation” (GO:0006412; 19 up-regulated genes; 1 down-regulated gene), “translational elongation” (GO:0006414; 8 up-regulated genes; 1 down-regulated gene), “rRNA processing” (GO:0006364; 11 up-regulated genes; 0 down-regulated genes), “ribosome biogenesis” (GO:0042254; 18 up-regulated genes; 1 down-regulated gene), “ribosomal large subunit export from nucleus” (GO:0000055; 2 up-regulated genes; 1 down-regulated gene) and “peptide biosynthetic process” (GO:0043043; 21 up-regulated genes; 1 down-regulated gene).

**Figure 4:**
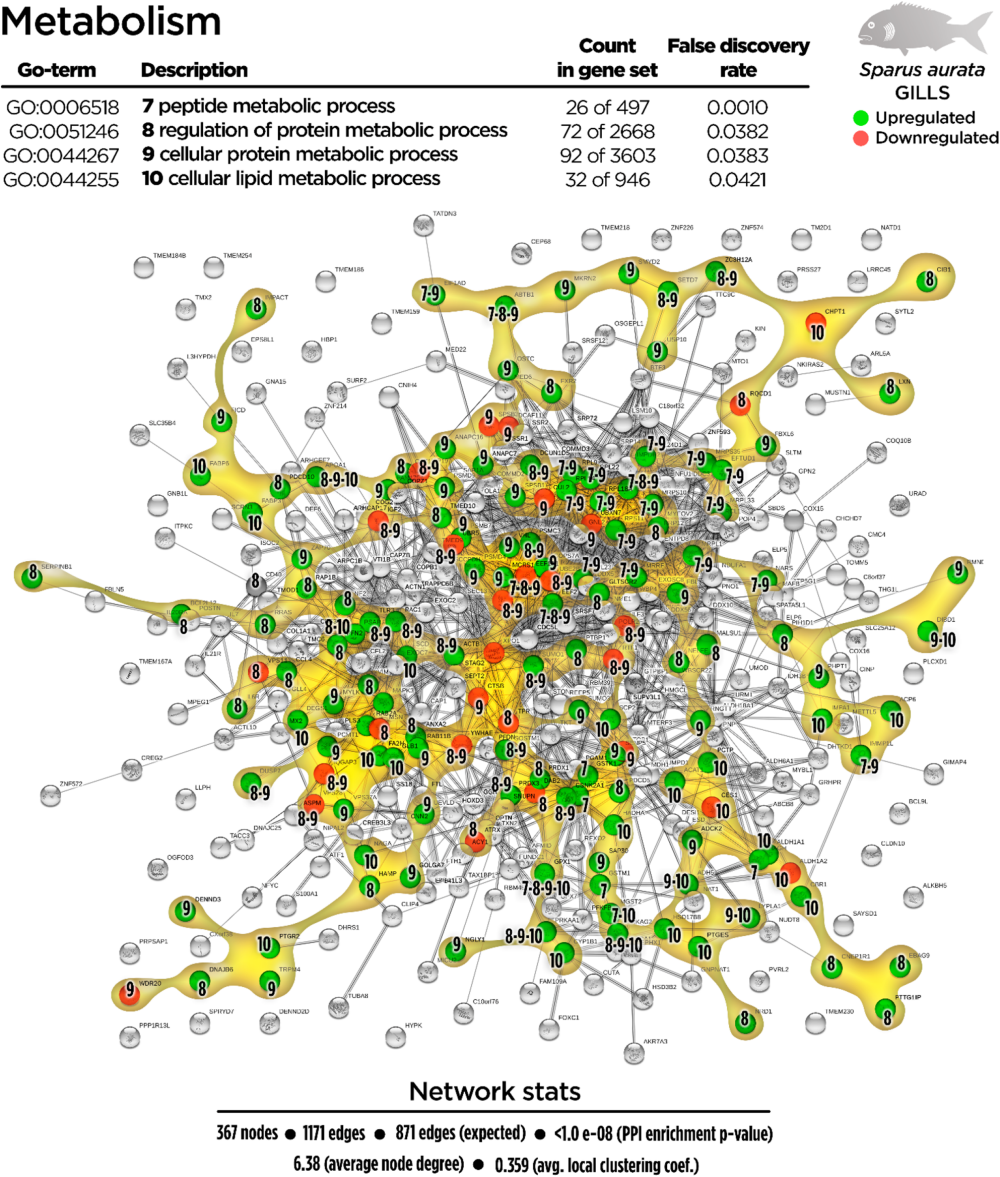
Metabolism-related Protein–Protein Interactions Network (PPI) network for the differentially expressed genes (DEGs) in the gills of juvenile gilthead seabream fed the garlic, carvacrol and thymol essential oils (EOs) supplemented diet (see also Supplementary Table 2). Nodes numbers (7–10) indicate the biological processes for each DEG represented. Gene Ontology (GO) definitions, count of DEGs within each biological processes and respective false discovery rate are described in the graphical figure legend. Green nodes represent up-regulated genes and red nodes represent down-regulated genes. Graphic keys and network stats are indicated in the graphical figure legend.

Metabolism-related processes were favoured by dietary EOs (132 up-regulated genes; 28 down-regulated genes). In agreement with biogenesis-related processes, the “peptide metabolic process” (GO:0006518; 28 up-regulated genes; 0 down-regulated genes) was also positively regulated in the gills of fish fed the EOs diet, among other processes such as the “regulation of protein metabolic process” (GO:0051246; 55 up-regulated genes; 18 down-regulated genes), “cellular protein metabolic process” (GO:0044267; 77 up-regulated genes; 17 down-regulated genes) and “cellular lipid metabolic process” (GO:0044255; 30 up-regulated genes; 3 down-regulated genes), which were expressed as shown in Fig. 4.

**Figure 5:**
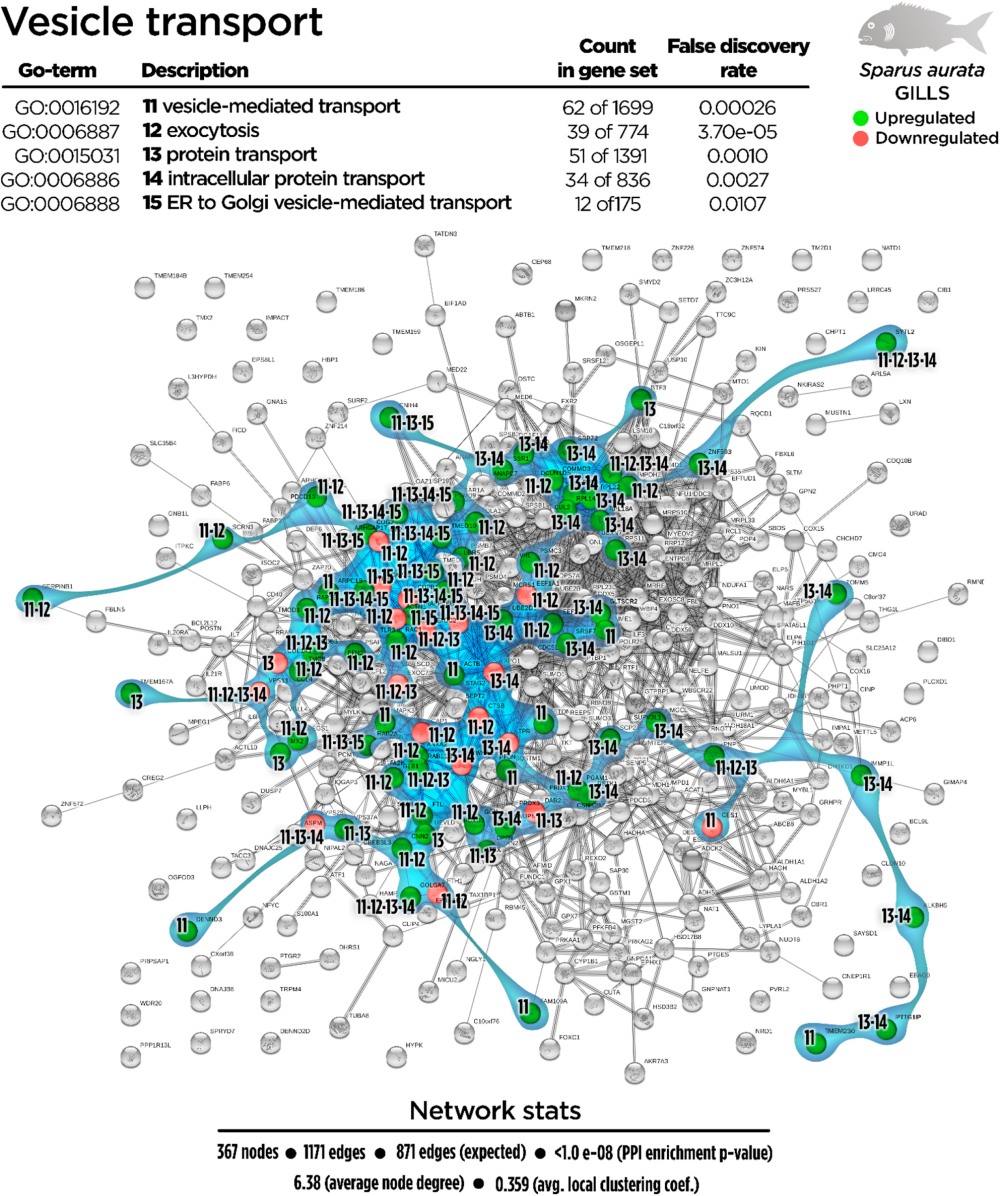
Vesicle-mediated transport Protein–Protein Interactions Network (PPI) network for the differentially expressed genes (DEGs) in the gills of juvenile gilthead seabream fed the garlic, carvacrol and thymol essential oils (EOs) supplemented diet (see also Supplementary Table 2). Nodes numbers (11–15) indicate the biological processes for each DEG represented. Gene Ontology (GO) definitions, count of DEGs within each biological processes and respective false discovery rate are described in the graphical figure legend. Green nodes represent up-regulated genes and red nodes represent down-regulated genes. Graphic keys and network stats are indicated in the graphical figure legend.

Genes associated with vesicular transport were positively regulated (72 up-regulated genes; 19 down-regulated genes) to dietary EOs (Fig. 5). Some of the GO were identified as representative such as “vesicle-mediated transport” (GO:0016192; 48 up-regulated genes; 15 down-regulated genes), “exocytosis” (GO:0006887; 30 up-regulated genes; 10 down-regulated genes), “protein transport” (GO:0015031; 44 up-regulated genes; 11 down-regulated genes), “intracellular protein transport” (GO:0046907; 31 up-regulated genes; 7 down-regulated genes) and “endoplasmic reticulum to Golgi vesicle-mediated transport” (GO:0006888; 9 up-regulated genes; 3 down-regulated genes).

**Figure 6:**
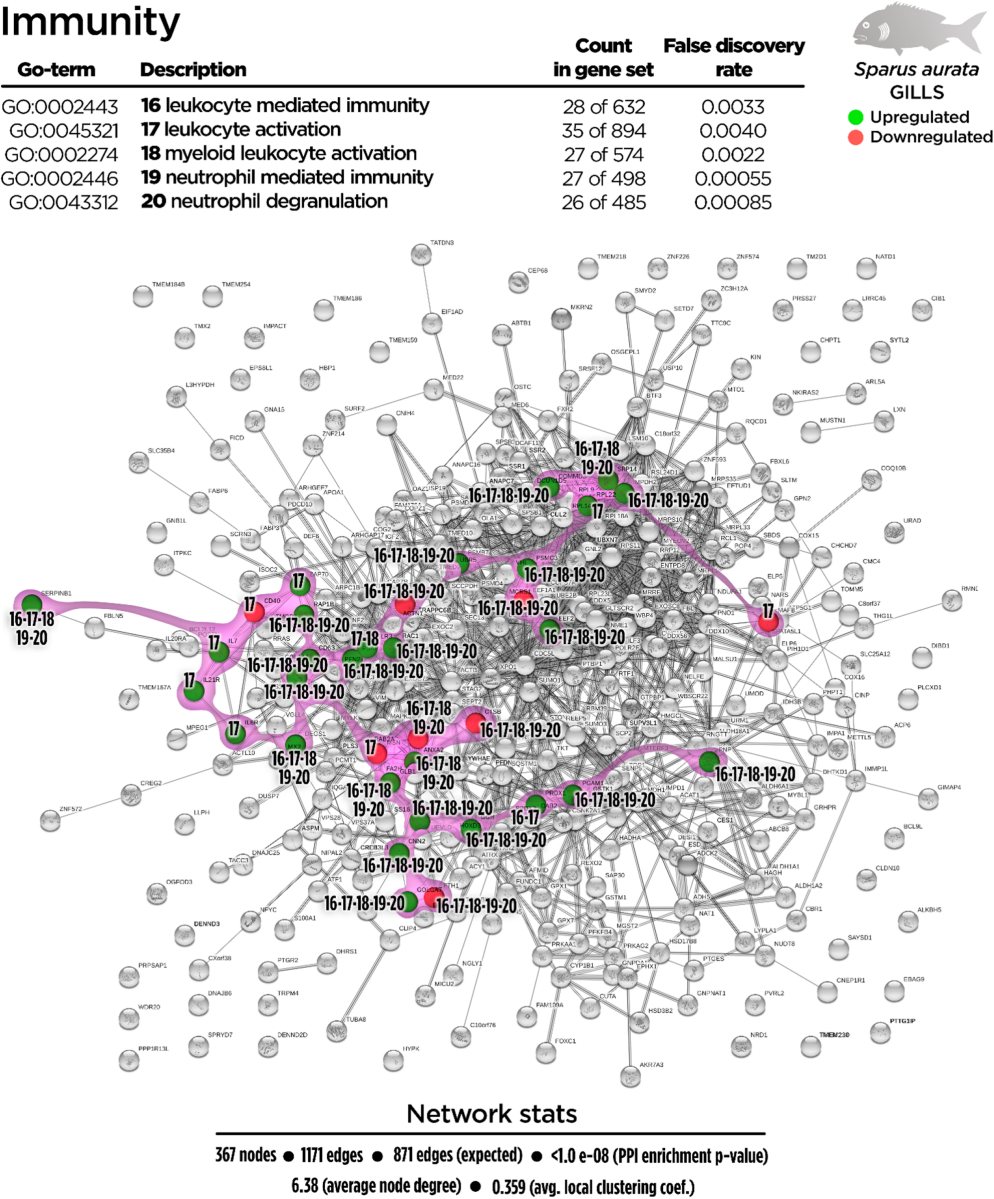
Immunity-related Protein–Protein Interactions Network (PPI) network for the differentially expressed genes (DEGs) in the gills of juvenile gilthead seabream fed the garlic, carvacrol and thymol essential oils (EOs) supplemented diet (see also Supplementary Table 2). Nodes numbers (16–20) indicate the biological processes for each DEG represented. Gene Ontology (GO) definitions, count of DEGs within each biological processes and respective false discovery rate are described in the graphical figure legend. Green nodes represent up-regulated genes and red nodes represent down-regulated genes. Graphic keys and network stats are indicated in the graphical figure legend.

**Figure 7:**
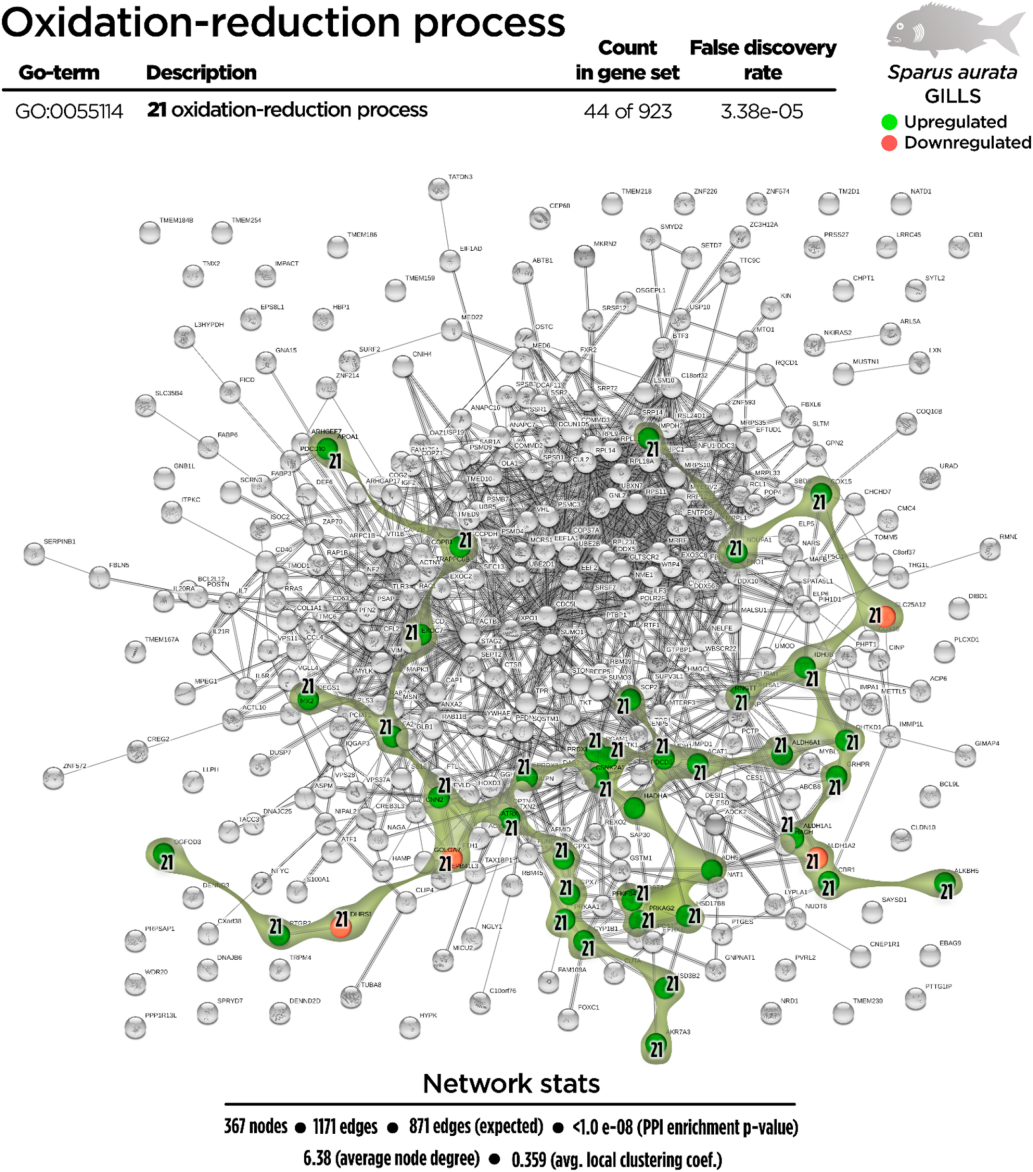
Oxidation–reduction Protein–Protein Interactions Network (PPI) network for the differentially expressed genes (DEGs) in the gills of juvenile gilthead seabream fed the garlic, carvacrol and thymol essential oils (EOs) supplemented diet (see also Supplementary Table 2). Nodes number (21) illustrate the oxidation–reduction biological process for each DEG represented. Gene Ontology (GO) definition, count of DEGs and respective false discovery rate are described in the graphical figure legend. Green nodes represent up-regulated genes and red nodes represent down-regulated genes. Network stats are indicated in the graphical figure legend.

Biological processes related to an immune effector response showed an important up-regulation response in gills (29 up-regulated genes; 8 down-regulated genes) of fish fed dietary EOs (Fig. 6). Some GO related with immunity were highlighted such as “leukocyte-mediated immunity” (GO:0002443; 24 up-regulated genes; 5 down-regulated genes), “leukocyte activation” (GO:0045321; 29 up-regulated genes; 8 down-regulated genes), “myeloid leukocyte activation” (GO:0002274; 23 up-regulated genes; 5 down-regulated genes), “neutrophil-mediated immunity” (GO:0002446; 23 up-regulated genes; 5 down-regulated genes) and “neutrophil degranulation” (GO:0043312; 22 up-regulated genes; 5 down-regulated genes). Particularly, the neutrophil-mediated immunity process shared 27 of 28 total regulated genes with the exocytosis process. A set of up-regulated genes related to anti-inflammatory response (*il7*, *il6r*, *il20ra* and *il21r*) were detected as mediators of immunity processes. Another relevant biological process positively affected by the dietary inclusion of EOs was the “oxidation–reduction process” (GO:0055114; 43 up-regulated genes and 4 down-regulated genes9 (Fig. 7).

### Histological organization and gill's histochemistry

No major differences in the histological organization of gills in fish from both experimental groups were observed (consult Feist^24^ for details on gill’s histological organization). Gills of fish fed the control diet showed no histochemical differences between the mucous cells (MCs) of primary and secondary gill lamellae. Nonetheless, the number of MCs was lower in the secondary lamellae (*ca*. 40 MCs mm^−1^) and more abundant in the epithelial and opercular areas, where they were intensely stained with most of histochemical and lectin techniques performed (Table 2). In brief, most of MCs from the control group contained abundant glycoproteins (GPs) with oxidizable vicinal diols groups (KOH-PAS), indicating the presence of neutral GPs, as well as in the epithelial cell layer due to the presence of secreted mucins. A scarce number of MCs contained GPs with O-sulphate esters (AB pH 1.0), whereas GPs with or without *O*-acyl sugars (KOH-PAS) were also registered in moderate or high amounts. Most MCs were strongly stained with PAS and diastase-PAS (absence of glycogen), whereas some of these cells containing neutral GPs also displayed a strong alcianophilia (AB pH 2.5), evidencing the presence of carboxylated GPs. Regarding lectins, MCs and the epithelial cell layers from the control group displayed from weak to moderate or strong affinity to several tested lectins (ConA, WGA, UEA-I, SNA and SBA) indicating the differential presence of Man/Glc, βGlcNAc, NeuNAc/sialic acids/NANA and GalNAc residues in mucins. The presence of GPs containing fucose residues was rare, being only evidenced in epithelial cell layers. In addition, the decreased reactivity after neuraminidase treatment before AB pH 2.5 and the presence of WGA and SNA lectins, indicated the occurrence of both non-acetylated and acetylated sialic acids in mucins.

**Table 2:** Histochemical properties of the mucous cells and the epithelium in gills from gilthead seabream fed a the control diet and the diet supplemented with a blend of garlic, carvacrol and thymol essential oils (EOs).

Diets	Epithelial cell layer (secreted mucins)		Mucous cells (non-secreted mucins)	
	Control	EOs	Control	EOs
**General histochemistry**				
Schiff	0	0	0	0
PAS	2	2	2	2
Diastase-PAS	2	2	2	2
KOH-PAS	2	2	3	3
Alcian-Blue (AB) pH 2.5	1	2	2	3
Alcian Blue pH 1	1	2	1	1
Alcian Blue pH 0.5	0–1	1	0	0
Neuraminidase-AB pH 2.5	0–1	1	1	1
**Lectin histochemistry**				
ConA	1	1	1	1
UEA-I	1	1	0	0
WGA	2	3	2	3
SNA	0–1	1	0–1	1
SBA	2	2	2	2

Results are expressed as the semiquantitative assessment of colour intensities by the scores of four independent observers: (0) negative; (1) weak; (2) moderate; (3) intense; and (4) very intense.

*PAS* Periodic Acid Schiff, *AB* Alcian Blue, *ConA* Concanavalin A, *UEA-I* *Ulex europeus* agglutinin, *WGA* Wheat germ agglutinin, *SNA* *Sambucus nigra* lectin, *SBA* Soybean agglutinin.

**Figure 8:**
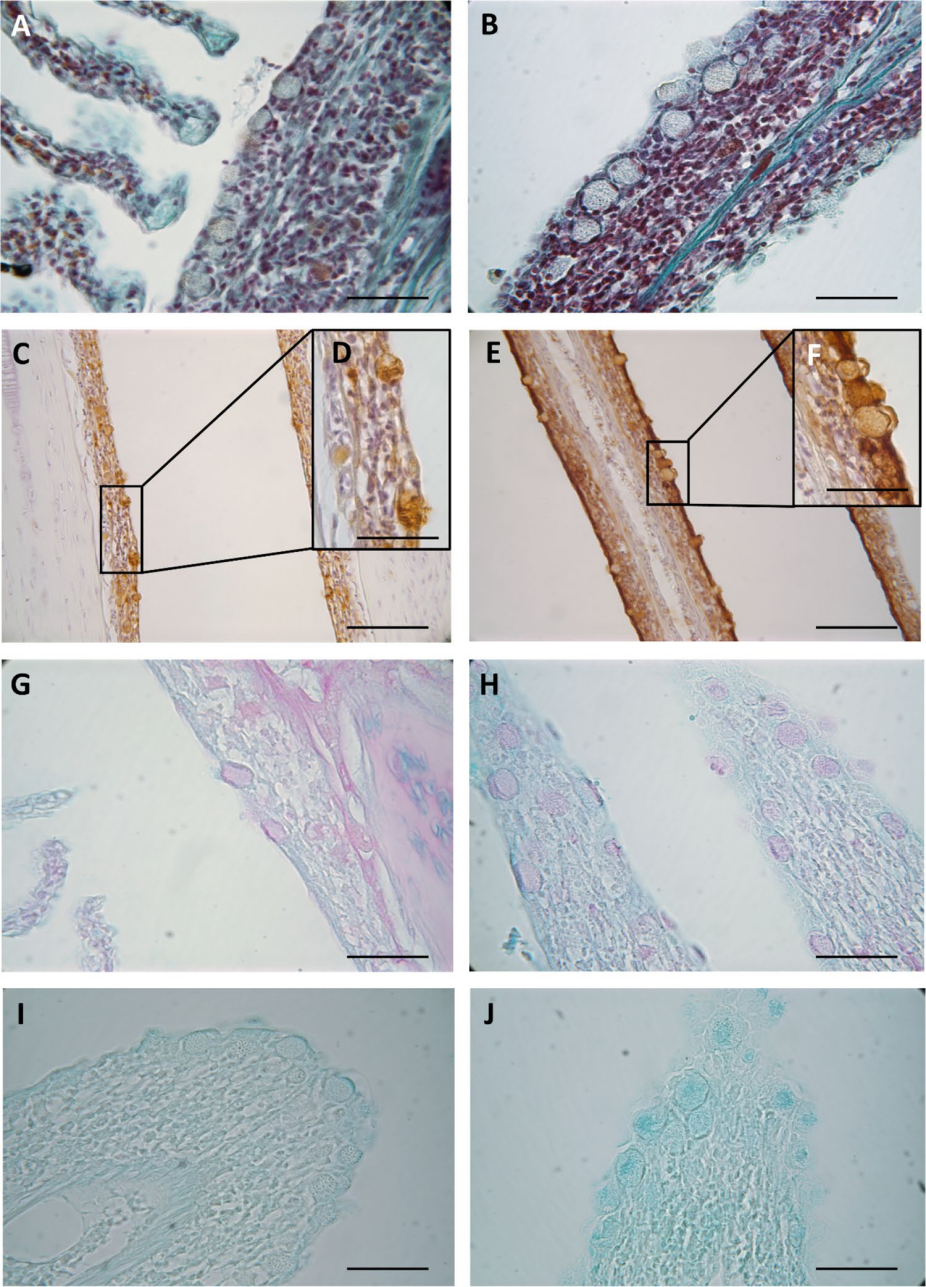
Histological sections from gills of gilthead seabream (*Sparus aurata*) fed the control diet (**A**, **C**, **D**, **G**, **I**) and the diet supplemented with a blend of garlic, carvacrol and thymol essential oils (EOs) (**B**, **E**, **F**, **H**, **I**). Histomorphological detail of mucous cells from control (**A**) and EOs (**B**) diets. Note the hypertrophy of mucous cells in fish fed the EOs diet (**B**) when compared to the size of mucous cells of the control group (**A**) (staining: H/VOF). Mucous cells content rich in *N*-acetyl-d-glucosamine and/or *N*-acetylneuraminic acid/sialic acid in fish fed control (**C**, **D**) and EOs (**E**, **F**) diets. Note increase in staining intensity in both epithelium and mucous cells from EOs diet (staining: WGA lectin). Presence of neutral glycoproteins in branchial mucous cells from control (**G**) and EOs (**H**) diets (staining: PAS). Carboxylated glycoproteins were also detected both in epithelium and mucous cells from control (**I**) and EOs (**J**) diets. Note increased in staining intensity in fish fed the EOs diet comparing to controls (staining: AB pH 2.5). Scale bars represent 25 (**A**, **B**, **D**, **F**, **G**, **H**, **I**, **J**) and 50 (**C**, **E**) µm.

Dietary EOs promoted modifications on the histochemical properties of MCs and their secretions. Although the number of MCs did not change, most of these secretory cells were hypertrophied (158.7 ± 9.9 µm^2^) when compared with the control group (91.7 ± 9.2 µm^2^) (Fig. 8). The most noticeable histochemical effect was the increase of carboxylated GPs (AB pH 2.5) containing sialic acid. In addition, residues of βGlcNAc and NeuNAc/sialic acids/NANA (WGA, SNA lectins) in the glycoconjugate contents of MCs and branchial epithelia were observed at higher intensity levels (Fig. 8, Table 2).

### Ectoparasite challenge

**Figure 9:**
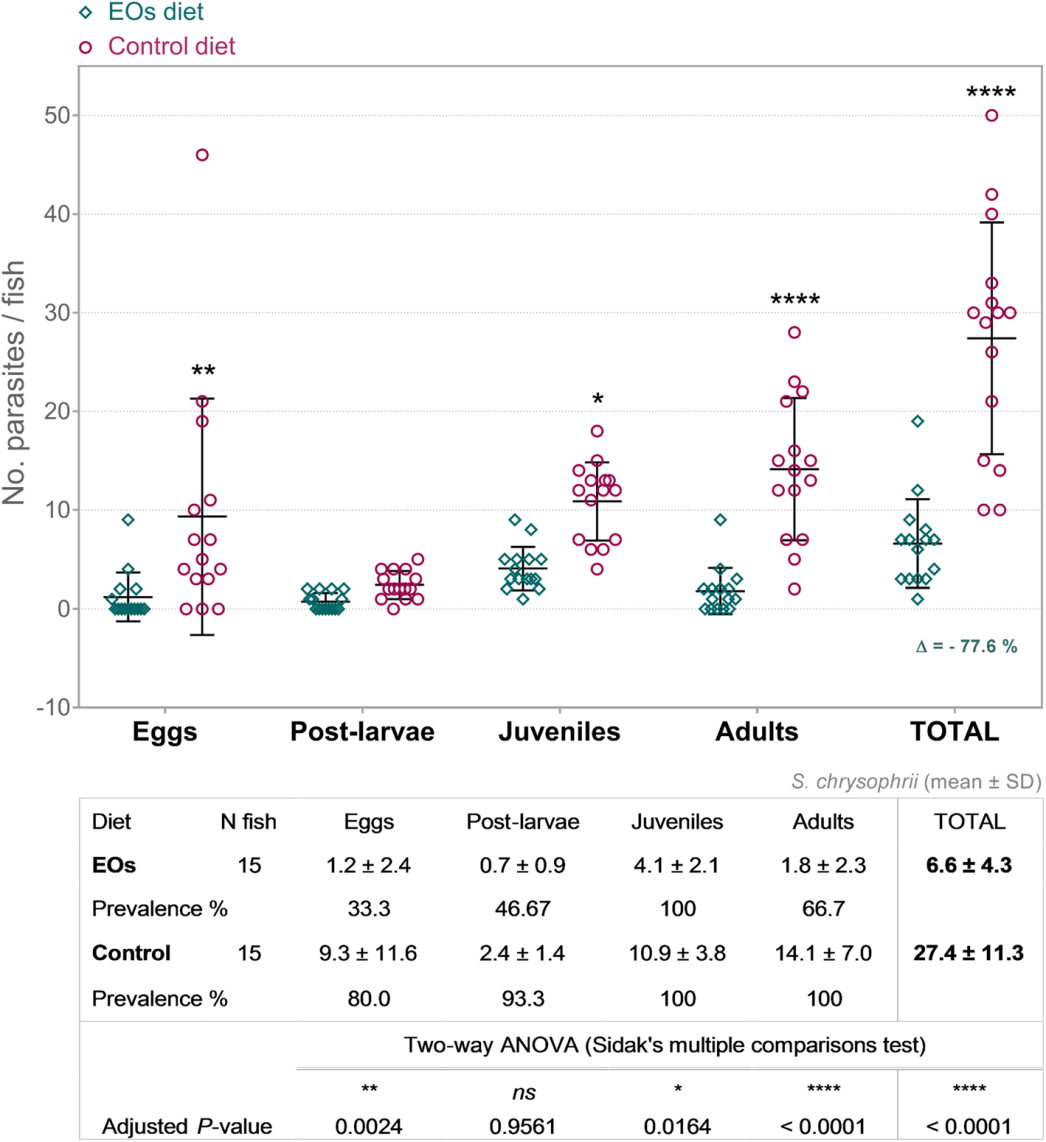
Abundance of *S. chrysophrii* parasites in fish fed with the control diet and the diet supplemented with a blend of garlic, carvacrol and thymol essential oils (EOs). Different ectoparasite developmental stages are represented according to their morphological characteristics: eggs, post-larvae (early juveniles with 2–4 pairs of clamps), juveniles and adults. The total load of the ectoparasite (TOTAL) and the percentage of total abundance decrease among experimental diets is also indicated (Δ). Circles and rhombus represent parasite counting per individual fish (n = 15); mean ± standard deviation are represented. Circles (pink): gilthead seabream fed with control diet; Rhombus (green): gilthead seabream fed with EOs supplemented diet. *, ** and **** indicate significant differences between dietary groups with corresponding adjusted P-values described.

Fish fed dietary EOs had a significantly lower number of total parasite intensity (6.6 ± 4.5 parasite fish^−1^) and abundance (6.6 ± 4.3 parasite fish^−1^) when compared to the control group (29.5 ± 15.1 and 27.4 ± 11.3 parasite fish^−1^, respectively) (P < 0.05; Fig. 9), resulting in a reduction of 77.6% of total parasite load. The number of eggs present in fish fed the EOs-diet in terms of intensity (3.6 ± 3.2 parasite fish^−1^) and abundance (1.2 ± 2.5 parasite fish^−1^) was lower than in the control group (10.8 ± 12.3 and 9.3 ± 12.0 parasite fish^−1^, respectively), and prevalence decreased from 80.0% in control group to 33.3% in the EOs group. The number of post-larvae present in fish fed the EOs-diet in terms of intensity (1.6 ± 0.5 parasite fish^−1^) and abundance (0.7 ± 0.9 parasite fish^−1^) was not significantly different than in the control group (2.4 ± 1.4 and 2.4 ± 1.4 parasite fish^−1^, respectively), and prevalence decreased from 93.3% in control group to 46.7% in the EOs group. However, the number of juvenile ectoparasites intensity and abundance was lower in fish fed the additive (4.1 ± 2.2 and 4.1 ± 2.1 parasite fish^−1^, respectively) in comparison to those fed the control diet (10.9 ± 4.0 and 10.9 ± 3.8 parasite fish^−1^, respectively), and prevalence was 100% for both experimental groups. The number of adults intensity and abundance was also lower in fish fed the additive (2.7 ± 2.4 and 1.8 ± 2.3 parasite fish^−1^, respectively) than in those fed the control diet (14.1 ± 7.2 and 14.1 ± 7.0 parasite fish^−1^, respectively), and prevalence decreased from 100% in control group to 66.7% in the EOs group.

## Discussion

The use of garlic, carvacrol and/or thymol EOs in functional aquafeeds has been tested and demonstrated both in vitro and in vivo for its effectiveness in fighting against bacterial and parasitic infections^2,25,26^. The anthelmintic properties of EOs and gill’s response to their dietary administration are poorly understood and available information is scarce. Hence, most of the existing studies are focused in the effects of the above-mentioned EOs on intestinal health^10,12,27,28^, as well as in their use in balneation treatments against parasitic organisms^15,16,29–31^. As far as we know, this is the first study describing the gill’s response in gilthead seabream to the administration of the above-mentioned EOs as a feed additive, as well as the mechanisms underlying its antiparasitic properties.

The dietary inclusion of microencapsulated garlic, carvacrol and thymol EOs did not affect fish growth. Contrarily, previous studies suggested them as growth promoters in several fish species^13,25,27,32–38^. Although the reasons explaining such differences are out of scope in this study, the utilization of different EO doses, administration length and diet formulation could be among the potential factors that may explain such results. Regardless of the fact that growth has been traditionally considered as one of the main end-points and/or key performance indicators for additive testing, some existing literature might not be trustworthy due to constrains in the reproducibility of the compounds studied^39^. Under this context, the use of natural plant-derivates or extracts may lead to variability and discrepancy of results, as opposed to the utilization of similar synthetic compounds, which favours the reproducibility and robustness of the studies. Moreover, SGR values of gilthead sea bream fed both control (2.1 ± 0.07% BW/day) and the EOs supplemented diet (2.03 ± 0.01% BW/day) compared favourably to those reported by Mongile et al.^40^ for this species also reared under summer conditions (1.5 ± 0.1% BW/day).

Gills are one of the main mucosal immune barriers in fish^22,41,42^, but they also represent an ideal site for the attachment of ectoparasites^43^, potentially inducing a host hypometabolic response, as suggested for *S. chrysophrii* infections^44^. In fact, gills are considered one of the most active tissues in the protein synthesis with a significantly high plasticity in protein metabolism^45^. Our transcriptional study revealed several biological processes associated to biogenesis and metabolic processes, including peptide biosynthesis, and protein and lipid metabolism that were predominantly up-regulated in the gills of gilthead seabream fed dietary EOs. Therefore, EOs would contribute to restore the gill's metabolic rate by increasing protein synthesis. Although gill’s protein turnover contribution is not significant to the whole body^45^, our transcriptional profile is in accordance with studies that have reported the significant influence of garlic^33,34^, carvacrol^46^ and thymol^13,35^ dietary administration on body and blood protein content upon the protein synthesis and metabolism. Collectively, our results provide new evidence for the biological activity of garlic, carvacrol and thymol, indicating these compounds also promote the protein synthesis and metabolism at mucosal level, particularly on gills (see text below); thus, increasing the overall activity of this tissue.

The vesicular trafficking processes are intrinsic to the secretory protein biogenesis^47^. Despite the previous evidence reporting an augment in the proteins synthesis^13,32–35,46^, the transcriptional regulation of vesicle-mediated transport by the dietary administration of garlic, carvacrol and thymol have not been described in fish to date. Genes involved in protein vesicle-mediated transport, such as the ER to Golgi vesicle-mediated transport process, were also positively regulated in the gills of fish fed dietary EOs, as for example the Secretion Associated Ras-related GTPase 1A (*sar1a*), the Rho Guanine Nucleotide Exchange Factor 7 (*arhgef7*), the Vesicle Transport Through Interaction With T-SNAREs 1B (*vti1b*) and some of the Rab Family of GTPases (*rab11b, rab2a*). The above-mentioned genes are known for their role in cellular trafficking pathways, like the RAB11-coding protein, which is recognized for its localization in recycling endosomes and its role in exocytic trafficking^48^. In vitro studies in mammal cells have associated garlic^49^, carvacrol and thymol to vesicle fusion and exocytic processes^50^. Therefore, we may infer that the machinery implied in the activation of biogenic processes observed by dietary EOs is inherent to the activation of processes of secretory protein translocation by vesicles.

As previously mentioned, vesicle trafficking and exocytosis are intimately related processes. In this way, genes like Rab GTPases participate in the regulation of the exocytosis membrane trafficking pathway^51^. In our gill’s transcriptional analysis, exocytosis was one of the most positively regulated processes by dietary EOs. This finding is especially relevant since exocytosis is recognized by its important role in the immune response participating in neutrophil function^52^, in the immunological synapses between cells^53^ and in the cell-mediated cytotoxicity^54^. Remarkably, the tested EOs also positively regulated immune-related biological processes by means of myeloid leukocyte immunity activation. Besides, this response appeared to be orchestrated by neutrophil population, since neutrophil mediated immunity and neutrophil degranulation processes were boosted by dietary EOs. In gilthead seabream, acidophilic granulocytes are functionally equivalent to higher vertebrates neutrophils^55,56^ being described as one of main phagocytes of this species^57,58^ occurring also in mucosal tissues^59–61^. Neutrophils’ granules are reputed for their antimicrobial, proteolytic and potential cytotoxic capacities^52,62^, which are synthetized during myeloid cell differentiation, comprising specific protein biosynthesis and the early formation of secretory vesicles^52^. Remarkably, we detected the presence of DEGs associated with protein biosynthesis, vesicular transport and exocytosis (as formerly discussed), which could be in their turn up-regulated due to the immunostimulatory effect of dietary EOs upon acidophilic granulocytes degranulation process. For instance, Mitogen-Activated Protein Kinase 3 gene (*mapk3*) resulted up-regulated in the gills of the EOs diet group, with representation both in protein and lipid metabolic and in the vesicle-mediated transport processes. Mitogen-activated protein kinases (MAPK) are known to be involved on signalling pathways of neutrophil functional response^63,64^. There is evidence of the activation of MAPK by ajoene (an organosulfur compound found in garlic) in the process of apoptosis of human cancer cells^65^. Despite of the evidences of an acidophilic granulocyte-mediated immune response stimulation induced by the bioactive compounds of the EOs tested, the exact bioactive compounds and accurate mechanisms involved in the alleged immunomodulatory and antiparasite effects of these EOs still needs to be deciphered when tested in separate.

In aquaculture relevant species, some studies reported an increase of blood neutrophil number after therapeutic balneation with EOs, which was also effective against monogeneans^29,66,67^. Neutrophils function involves the interplay of many different receptors, ion channels and signalling pathways, such as changes in intracellular Ca^2+^ levels, for instance^68^. Accordingly, garlic EO organosulfur compounds were recently demonstrated to activate human neutrophil functional activity through the activation of Ca^2+^ flux^64^, whereas ajoene and allicin were described as potent inhibitors of neutrophil ROS production^64^. Similar results were also attributed to thymol redox properties^69^, whereas in fish, dietary carvacrol (0.05%) significantly reduced leukocyte ROS release in seabass^11^. Although neutrophils and other circulating leukocytes have a critical role in the innate immune defence against pathogens, such helminth parasites^70^ including monogeneans^71^; and that acidophilic granulocytes have been particularly identified in gilthead seabream gill's response to ectoparasite infections^59^; its activity comprises a significant tissue damage associated to the ROS released during the inflammatory process. For instance, it was proposed that gilthead seabream may control *S. chrysophrii* infection through ROS action produced by immune cells. Although parasite evasion mechanisms were also suggested, this response may potentiate secondary infections if it is not properly controlled^72^. Although it might seem contradictory, the sustenance of self-protective antioxidant mechanisms is vital for the correct functioning of the immune system, preventing oxidative damage by ROS that escort leukocyte activity; particularly in neutrophil-mediated immune response^73^. Outstandingly, these antioxidant properties were also highlighted in the transcriptomic profiling of the gills in fish fed dietary EOs, where several genes involved in oxidation–reduction processes were positively regulated. Aquafeeds containing natural garlic (4%), garlic powder (3.2%) and garlic oil (0.25%) promoted glutathione peroxidase, superoxide dismutase and catalase activities in tilapia serum and liver^33^. In seabass, after cadmium-induced toxicity, the up-regulation of genes coding for these enzymes in the liver improved the antioxidant capacity of individuals fed a diet containing garlic powder (2%)^74^. Similarly, an increased antioxidant activity in rainbow trout fillet was associated to dietary carvacrol (1.2%) or thymol (0.6%)^10^, and in channel catfish, a commercial product containing *O. heracleoticum* EO (0.05%) enhanced plasma antioxidant activity^32^. In accordance with the above-mentioned studies, our transcriptomic data showed that dietary EOs promoted the up-regulation of Glutathione Peroxidases (*gpx1*, *gpx7*) and Glutathione S-transferases (*gstm1, gstk1, mgst2*). Additionally, Mitochondrial Thioredoxin (*txn2*), a key antioxidant protein that participates in the removal of ROS and cytotoxicity^75,76^, was also up-regulated by dietary EOs. In our study Peroxiredoxins 1 and 3 (*prdx1, prdx3*) were also up-regulated. Additionally, the up-regulation of the Epoxide Hydrolase 1 (*ephx1*) was promoted by dietary EOs; this enzyme has a detoxifying function, playing an important role in cellular and organ defence against exogenous toxicity compounds^77^. Thus, it appears that tested EOs may exert an important antioxidant action to counteract the impact of the high amounts of ROS released by the previously referred stimulation of acidophilic granulocyte’s activity, evidencing the importance of their joint supplementation.

After a proinflammatory phase starred by neutrophils and their ROS production, the induction of a resolution phase is mandatory to prevent persistent harmful inflammation and oxidative stress in the host cells^76^; thus, the activation of the anti-inflammatory response is needed to minimize such side-effects^78^. Therefore, the leukocyte activation together with the up-regulation of a repertoire of anti-inflammatory cytokines (i.e. *il7, il6r, il20ra* and *il21r*) is representative of this intimate coordination between both processes, suggesting also an added anti-inflammatory response triggered by the dietary EOs. The modulation of cytokine expression and immune cell stimulation are the mechanisms attributed to the biological activity of garlic compounds^79^. In addition, there are also processes associated to the anti-inflammatory bioactivity of carvacrol and thymol^80,81^. For instance, carvacrol was described as an inhibitor of human neutrophil elastase^82^. In our study, the Serpin Family B Member 1 (*serpinb1*), a protein that inhibits neutrophil elastases protecting tissue from damage at inflammatory sites, was also up-regulated by dietary EOs, corroborating its anti-inflammatory properties. In fish, the up-regulation of inflammatory cytokine genes was observed in the gut of tilapia fed dietary garlic powder (1.0%)^17^. In juvenile gilthead seabream, the dietary inclusion of a commercial encapsulated combination of carvacrol and thymol EOs (0.01%) resulted in an enhancement of the intestine absorptive capacity that was attributed to the induction of anti-inflammatory and anti-proliferative gene markers^12^. The same commercial additive also demonstrated an immunostimulatory effect in juvenile hybrid tilapia^28^.

Gills of gilthead seabream infested with *S. chrysophrii* showed an over-expression of apoptosis, cell proliferation and inflammation processes^44^. Thus, under an infective process, the combination of the pro- and anti-inflammatory mechanisms is also required for a successful pathogen eradication^78^. Although our transcriptomic profiling of gills was conducted at the end of the nutritional trial before fish being exposed to the ectoparasite, the build-up of a local former anti-inflammatory response induced by dietary EOs might delay the effect of the inflammatory outcome associated to *S. chrysophrii*; thus, potentially reducing tissue damage. This regulation observed in our study could enhance tissue protection and regeneration mechanisms involved in gill's responses against ectoparasites, not only by the stimulation of an inflammatory response, but also by means of antioxidant and anti-inflammatory processes.

One of the common characteristics of mucosal-associated lymphoid tissues, including the GIALT, is the presence of mucus-secreting cells^42^. The main components of mucus are mucins, high molecular weight glycoproteins (GPs) with numerous carbohydrate chains *O*-glycosidically linked to a protein core. Both commensal and pathogenic microorganisms^83–85^, and likewise monogenean parasites^86,87^, use this mucosal GPs as receptors for their attachment. A high variety of mucin oligosaccharides forms an extensive repertoire of attachment sites with different carbohydrate specificities^88^. Peculiarly, sialic acids and related saccharide residues can serve as receptor sites for binding exogenous macromolecules such as those of bacterial, viral or parasitical aetiology, playing an important role as “decoy” for pathogens, in such a strategy where the sialylated mucins are shed with the anchored sialic-binding pathogen^89,90^. Under parasite infections, qualitative changes of fish mucus occur, mainly in the mucin glycosylation pattern^91–93^. Thus, increases in sialic acid and *N*-acetylglucosamine terminal in mucins are described in as a host defence against helminths^94^. Nevertheless, many pathogens and parasites have evolved to disrupt the mucin barrier; for instance, several digestive mucins were down-regulated and a significant reduction of MC positive for sialic acid was observed in gut-parasitized gilthead seabream^95–97^.

In our study, it is relevant to highlight an increase of carboxylated and/or sulphated GPs containing sialic acid and of *N*-acetylglucosamine/β-d-GlcNAc residues in gills of fish fed dietary EOs. Neutral GPs lubricate, facilitate gas exchange and regulate the acidity of mucous secretions, whereas acid carboxylated and sulphated GPs are more viscous, a characteristic associated to their antibacterial and antiparasitic properties^85,98–100^. The higher presence of acid GPs coupled with the increase in sialic acid and *N*-acetylglucosamine in mucins may be associated to an enhanced protection against *S. chrysophrii* attachment. Furthermore, MCs hypertrophy observed in gills of fish fed dietary EOs indicated a potentiation of the mucosal secretion and renewal, boosting its protective function. These results may be a consequence of the synergy between garlic, carvacrol and thymol EOs, since such histochemical differences and antiparasitic effects were not observed when garlic was tested by separate (Supplementary Information 1). Transcriptomics revealed that ALG9 Alpha-1,2-mannosyltraferase (*dibd1*), which is involved in *N*-glycan biosynthesis^101^, was positively regulated by dietary EOs, which is one of the most common post-translational modifications of proteins^102^. Additionally, we found an up-regulation of *O*-Sialoglycoprotein Endopeptidase like 1 (*osgepl1*), whose enzyme is for long commercially used and recognized for its mucin-degrading activity, and which increase might indicate an enhance of the proteolytic mucin degradation^103^, which is characteristic of the host GPs “shedding” defence mechanism. In this way, although not evidenced among the main representative processes in our gill's transcripteractome outcome, the regulation of some of pathways such as translational elongation, peptide biosynthesis, cellular protein metabolism, intracellular protein transport, and exocytosis-related processes, might be correlated with those of the mucosal surface.

Altogether, transcriptomic results suggest that dietary EOs may be promoting the synthesis and release of GPs detected at histological level, which might have a beneficial functionality on the gills and potentially reducing the *S. chrysophrii* attachment to gill’s surface. Accordingly, gilthead seabream fed dietary EOs showed a reduction of 78% of total parasite load when compared with the control group, with a decrease in the prevalence of most of the parasitic developmental stages as well. Garlic is known for its wide-spectrum of antimicrobial activity that is attributed to allicin and ajoene, which exert multiple inhibitory effects on thiol-dependent enzymatic systems^104^. Similarly, garlic-based treatments demonstrated to be particularly effective in the fight against monogeneans and other parasites^18,105,106^. Farmed barramundi fed diets containing a garlic extract (50 and 150 mL kg^−1^) for 30 days showed a reduction of *Neobenedenia* sp. oncomiracidia stage^19^. Similarly, garlic extract administered by balneation (0.76 and 15.2 µL L^−1^ allicin concentration) had also antiparasitic properties towards *Neobenedenia* sp.^22^. In guppy, both diet (10 and 20% garlic powder) and bath (7.5 and 12.5 mL L^−1^ garlic extract; 1 g L^−1^ fresh crushed garlic) garlic-based treatments were successful against *Gyrodactylus turnbulli*^20^. The application of garlic bath treatments (3 ppt garlic oil; 300 mg L^−1^ crushed garlic cloves) resulted effective against *Trichodina* sp. and *Gyrodactylus* sp. in Nile tilapia^16^. However, an in vitro treatment with a water–ethanol extract of garlic tested at different dilutions (1:10, 1:50 and 1:100) showed no overall antiparasitic effect on *Neobenedenia* sp.^107^. The instability of free organosulfurs may lead to contradictory results in terms of the efficiency of garlic extracts and doses against monogenean parasites. These results highlight the benefits of encapsulating this type of compounds for dietary administration, since this process ensures their dietary stability, preventing inopportune interactions with the host and environment, and allowing their proper delivery in the gastrointestinal tract^4^.

Concerning carvacrol and thymol, several bath treatments with different EOs proved to be effective anthelmintics^29–31^. The antiparasitic action of carvacrol against protozoans in chum salmon was associated to its presence in the skin of fish fed a diet supplemented with oregano EO at 0.02%^109^. Thymol was also demonstrated to have antiparasitic effects against the protozoan *Leishmania* sp.^108^ and sheep gastrointestinal nematode helminths^109^. Nonetheless, there are few studies that accurately describe the antiparasitic effect of dietary phytochemicals against monogeneans in fish species; thus, the pathways and mechanisms of their action are not clear yet^110^. Regardless of this fact, present transcriptomic data from gills at the end of the nutritional trial provided the base line knowledge for deciphering the antiparasitic role of the tested EOs. While fish immunity against monogenean parasites is certainly multifactorial and innate factors seem to dominate the first response against this parasites^111–113^, the participation of the mucosal adaptive factors, such as B-lymphocytes^42,114^, immunoglobulins^115–117^ or even specific antibodies^118–120^, could be critical for longstanding parasite suppression^71,86^. Responses against helminth parasites also include the expression of classical effector type 2 cytokines that will signal the recruitment of inflammatory cells and induce goblet cell hyperplasia leading to mucus production^121^. Nevertheless, the specific mechanisms modulated during the parasitic challenge were not evaluated in this work, thus further studies are needed to determine if a type 2 immune response is also implicated in the success of the diet-induced antiparasitic response. Studies focused on identifying the bioactive compounds responsible for the gill's response observed in the present work are currently in progress.

Concluding, we showed that the administration of a microencapsulated feed additive containing garlic, carvacrol and thymol EOs in gilthead seabream promoted the activation of protein biosynthetic processes in gills. These biogenic processes are highly related to the translation of mRNA into proteins, which in turn are actively mobilized by vesicular transport and exocytosis. This mechanism activates effector leukocytes like acidophilic granulocytes. The immune response promoted by dietary EOs is also supported by the active control of oxidation–reduction processes, the building of an anti-inflammatory local response and the changes in the histochemical properties of mucins produced by branchial MCs. The overall results of our study highlighted the main biological processes induced by this dietary EOs that might be responsible for the later antiparasitic response observed in gills against *S. chrysophrii*. The notorious effect of the tested dietary EOs suggests its application as preventive and active treatment for this particular ectoparasite and a promising alternative treatment for other infections, although further evaluation is needed in order to validate this hypothesis.

## Methods

### Diets

A basal diet (46% crude protein; 18% crude fat; energy: 21.5 MJ kg^−1^) was formulated (Table 3) to meet the nutritional requirements of gilthead seabream under summer conditions^40^. The experimental diet contained a microencapsulated additive at 0.5% composed of a blend of garlic, carvacrol and thymol synthetic EOs (AROTEC-G, TECNOVIT-FARMFAES S.L., Spain). Both extruded diets (pellet size: 2 mm) were manufactured by SPAROS Lda. (Portugal).

**Table 3:** Formulation and proximate composition of the basal diet used during the nutritional assay.

Ingredients	Basal diet (%)
Fishmeal 70 LT FF Skagen	20.0
Fishmeal CORPESCA Super Prime	10.0
CPSP 90	2.5
Squid meal	2.5
Soy protein concentrate (Soycomil)	5.0
Wheat Gluten	5.0
Corn gluten	8.0
Korfeed 60	4.5
Soybean meal 48	8.0
Rapeseed meal	4.0
Sunflower meal	3.0
Wheat meal	7.0
Whole peas	2.5
Fish oil—COPPENS	9.0
Soybean oil	1.5
Rapeseed oil	2.5
Vitamin and mineral Premix PV01	2.0
Soy lecithin—Powder	2.0
Antioxidant powder (Paramega)	0.4
Dicalcium phosphate	0.6
TOTAL	100.0
**Proximate composition, % in dry basis**	
Crude protein	46.2
Crude fat	18.4
Gross energy	21.5

### Fish rearing and nutritional assay

**Figure 10:**
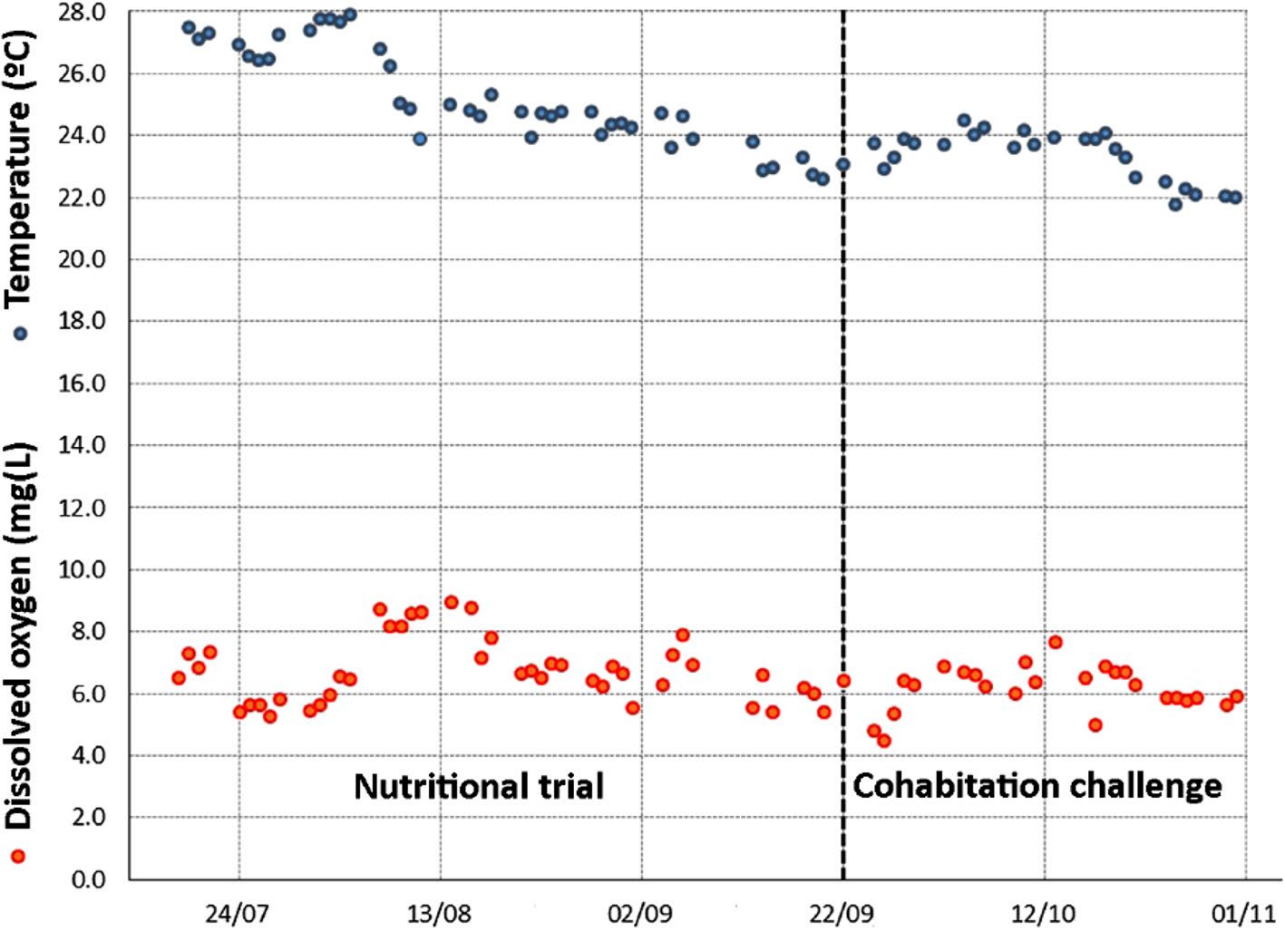
Daily mean values for water temperature (°C) and dissolved oxygen (mg L^−1^) in experimental tanks along the nutritional and cohabitation trial conducted in order to evaluate the effect of a diet supplemented with a blend of essential oils (garlic, carvacrol and thymol) against an ectoparasite infestation by *Sparicotyle chrisophrii*. Daily data are computed using the individual values for each experimental tank (n = 6); no data for weekends or holidays are reported since these variables were not manually measured during these days.

Gilthead seabream (body weight, BW = 5.0 ± 0.2 g; mean ± standard deviation) were obtained from Piscicultura Marina Mediterránea SL (Spain). After 105 days, 150 fish (BW = 40.3 ± 0.1 g) were distributed in six 450 L tanks connected to an IRTAmar recirculation system under open-flow water regimen and natural photoperiod (geographical coordinates ETRS89 system = 0.660418 E, 40.627516 N). Water temperature (24.6 ± 1.6 °C; range 21–28 °C; Fig. 10), oxygen (7.0 ± 1.7 mg L^−1^; > 80% saturation) (OXI330, Crison Instruments) and pH (7.5 ± 0.01) (pHmeter 507, Crison Instruments) were daily controlled. Salinity (35‰) (MASTER-20 T; ATAGO Co. Ltd), ammonia (0.13 ± 0.1 mg NH_4_^+^ L^−1^) and nitrite (0.18 ± 0.1 mg NO_2_^−^ L^−1^) levels (HACH-DR9000 Colorimeter, Hach) were weekly monitored. Data on water temperature and oxygen levels during the full experiment are depicted in Fig. 10.

The nutritional trial was run in triplicate (initial density = 2 kg m^−3^; 25 fish tank^−1^) during the summer and early autumn period. During 104 days, fish were fed both diets twice per day at apparent satiation (feeding rate = 3.0% of the stocked biomass). Fish were individually weighed at the beginning (end of July) and at the end of the nutritional assay (65 days, end of September), and at the end of the cohabitation/challenge period (104 days, end of October; see “Cohabitation challenge with *S. chrysophrii*”). At 65 days, four fish were selected from each tank, euthanized (MS-222, Sigma-Aldrich) and their second gill arch (right side) sampled for histological and transcriptomic analyses.

### Transcriptional analysis

#### RNA isolation and quality control

Gills were fixed in RNAlater (Invitrogen, Thermo Fisher Scientific), incubated overnight (4 °C), and stored at − 80 °C. Approximately 20 mg of whole filaments of the gill lamellae medial portion were removed from the bone (~ 1 cm longitudinally close to the bone) and homogenized with a cell disrupter. Total RNA was extracted (n = 8 fish per diet) using the RNeasy Mini Kit (Qiagen, Germany) and eluted (final volume = 35 μL) in nuclease-free water and treated with DNAse (DNA-free DNA Removal Kit; Invitrogen). Total RNA concentration and purity were quantified using a Nanodrop-2000 (Thermo Scientific) and stored at − 80 °C. Prior to hybridization, samples were diluted to 133.33 ng µL^−1^ and checked for integrity (Agilent 2100 Bioanalyzer; Agilent Technologies, Spain). RNA samples (RIN value > 8.5) were pooled in three sets per diet (two sample pools with n = 4 fish each; and a third pool combining 1:1 of the former pools).

#### Microarray design, hybridization and analysis

Transcriptional analysis was done using the Aquagenomics *Sparus aurata* oligonucleotide microarray v2.0 (4 × 44 K) (SAQ) platform. Platform information and transcriptomic raw data are available through Gene Expression Omnibus (GEO) at NCBI (accession numbers GPL13442 and GSE144055, respectively).

Analyses were conducted using a one-color RNA labelling (Agilent One-Color RNA Spike-In kit; Agilent Technologies). Total RNA (200 ng) from each sample pool were reverse-transcribed with spike-in. Total RNA was used as template for Cyanine-3 labelled cRNA synthesis and amplification kit (Quick Amp Labelling kit). cRNA samples were purified using the RNeasy micro kit (Qiagen). Dye incorporation and cRNA yield were checked (NanoDrop ND-2000); Cy3-labeled cRNA (1.5 mg) with specific activity > 6.0 pmol Cy3/mg cRNA were fragmented (60 °C, 30 min), and then mixed with the hybridization buffer (Gene Expression Hybridization kit, Agilent Technologies), and hybridized (65 °C, 17 h) to the array (ID 025603, Agilent Technologies). Washes were conducted using Gene expression wash buffers, stabilization and drying solutions. Microarray slides were scanned (Agilent G2505B Microarray Scanner System) and spot intensities and other quality control features extracted (Agilent Feature Extraction software version 10.4.0.0).

#### Transcripteractome

The complete map of interactions (interactome) was obtained from differentially expressed genes (DEGs) obtained in the microarrays-based transcriptomic analysis, the so-called transcripteractome^122^. The Search Tool for the Retrieval of Interacting Genes (STRING) (https://string-db.org) was used^123^. Protein–protein interaction (PPI) network of DEGs was conducted with a high-confidence interaction score (0.9) using *Homo sapiens* dataset. Genecards^124^ and Uniprot^125^ databases were used to confirm match of gene acronym between *H. sapiens* and gilthead seabream. Gene ontology (GO) enrichment analysis for DEGs was performed with STRING (P < 0.05).

### Histochemistry of the branchial tissue and mucous cells

The second gill arch from the right side was dissected from four fish per tank (n = 12 per diet) and fixed in 10% neutral-buffered formalin. After dehydration, tissues were embedded in paraffin and sectioned (3–5 μm thick). Two sections were stained with haematoxylin–eosin; the rest were used for evaluating the histochemical properties of branchial epithelia and mucous cells. These histochemical techniques were performed: Schiff, Periodic Acid Schiff (PAS), diastase-PAS, KOH-PAS, Alcian Blue (AB) pH 0.5, 1.0 and 2.5, and neuraminidase-AB pH 2.5 Underwood^126^. For characterization of glucidic residues bound to glycoconjugates, these horseradish peroxidase (HRP) conjugated lectins (Sigma-Aldrich, Spain) were used: *Canavalia ensiformes*/ConA (Mannose and/or Glucose), *Triticum vulgaris*/WGA (*N*-acetyl-d-glucosamine and/or *N*-acetylneuraminic acid, NeuNAc/sialic acid/NANA), *Ulex europeus*/UEA-I (l-Fucose), *Sambucus nigra*/SNA (NeuNAc/sialic acid/NANA) and *Glycine max*/SBA (α-*N*-acetyl-d-galactosamine). Sections were treated with 0.3% H_2_O_2_ for 10 min (endogenous peroxidase inhibition) in Tris-buffered saline (TBS; pH 7.2) and incubated for 30 min at RT in HPR-lectin conjugated (20 µg mL^−1^) dissolved in TBS. After three TBS washes, peroxidase activity was visualized with TBS containing 0.05% 3,3′-diaminobenzidine tetrahydrochloride and 0.015% H_2_O_2_. Sections were washed in running water (10 min), dehydrated, cleared and mounted. Controls were described as in Sarasquete, et al.^84^. Histochemical results were visualized under a light microscope (Leitz diaplan) and manually registered on a table. Results were expressed as the semiquantitative assessment of colour intensity scores [0, negative; 1, weak; 2, moderate; 3, intense; 4, very intense] from four independent observers (Supplementary Information 2). Mucous cell count was determined in four different gill regions and their number expressed per length unit of the basal lamina (1 mm), according to Yamamoto et al.^127^. The size of mucous cells was measured as the surface area (expressed as µm^2^) of 20 randomly selected cells of each gill section using a Spot (5.2) imaging software.

### Cohabitation challenge with *S. chrysophrii*

#### Establishment of a fish donors’ stock

**Figure 11:**
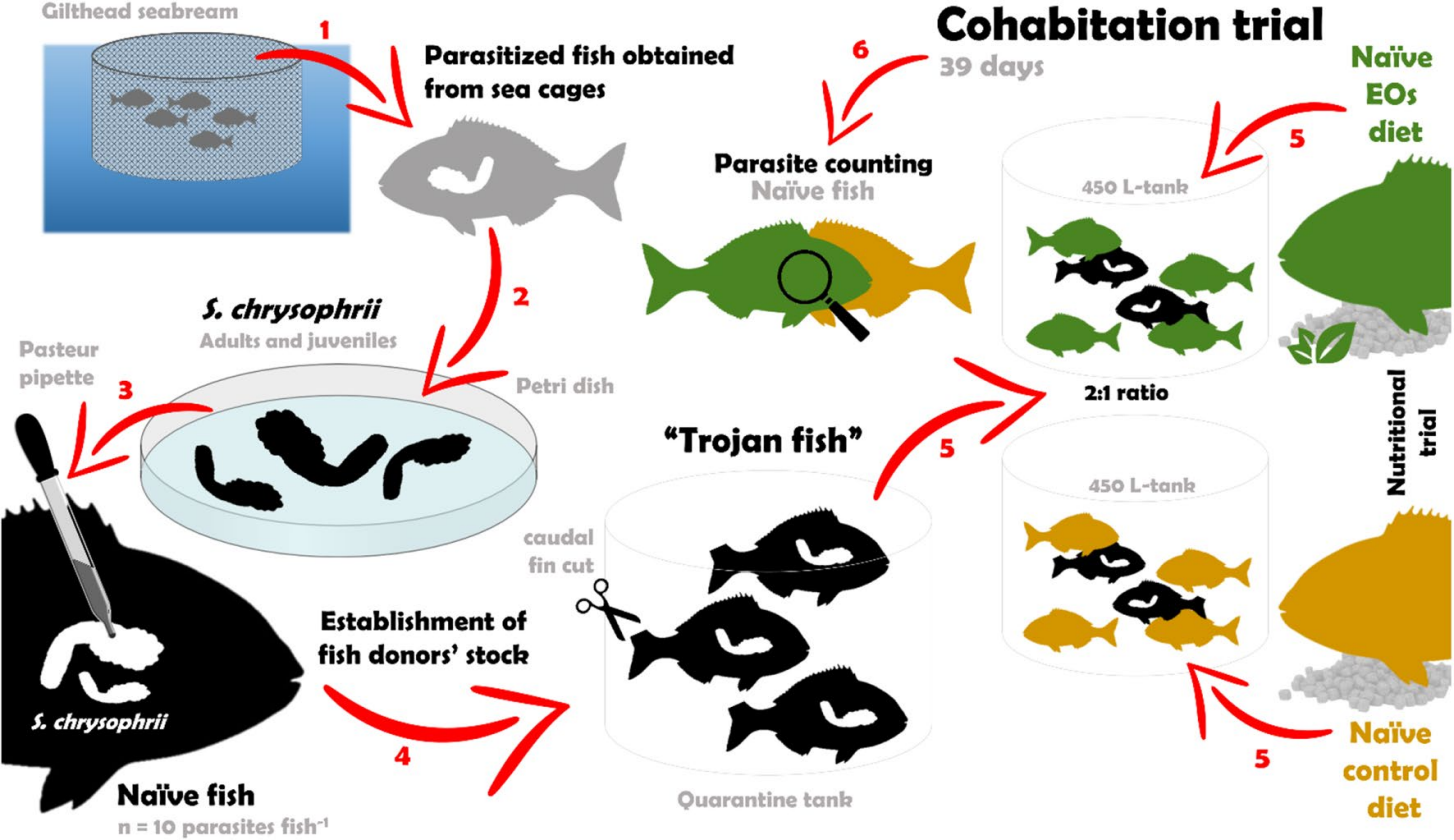
Schematic representation of the cohabitation trial set-up (see “Methods” section for details of each step).

Parasitized fish were obtained from sea cages of a private fish farm (data not provided for confidentiality purposes) and transported to IRTA facilities. Then, part of the parasitized fish were sacrificed, gills dissected and *S. chrysophrii* (juveniles and adults) placed in petri dishes with sea water until their inoculation in healthy fish. This strategy was chosen to avoid the presence of other branchial parasites^128^. Naïve fish previously anesthetized (MS222, 20 ppm) were infested on the left branchial lamellae with *S. chrysophrii* (n = 10 parasites fish^−1^) with a Pasteur pipette. The presence of the parasite in gill lamellae was visually checked to confirm its successful attachment. In case the parasite did not properly attach to the gill lamella, it was rescued from the water and the infestive process repeated. Fish successfully infested were selected as “Trojan fish” for the cohabitation challenge, transferred to a quarantine tank and periodically sacrificed to confirm and estimate the number of parasites. A graphical summary of this process is presented in Fig. 11.

#### Cohabitation trial and parasite counting

The potential beneficial effect of the blend of EOs in infested fish was tested in a cohabitation challenge with *S. chrysophrii* (Fig. 11). For this purpose, 27 fish from each nutritional group (9 fish from each replicate tank; naïve fish) were randomly selected and moved to 450 L-tanks (n = 27 fish per tank; 1 tank per each diet administered). Each individual fish was considered as an experimental unit to meet the 3Rs principles of animal experimentation^129^. Therefore, welfare issues were assessed in agreement with good culture practices, where population density was established within each tank without jeopardizing the infestation procedure and its efficiency. Trojan (infested) fish (BW = 110.5 ± 6.6 g) were randomly selected from the parasitized fish tank and the tip of their caudal fin cut to distinguish them from naïve ones. Given the low infestation rate on the Trojan fish, a 2:1 ratio (27 naïve: 14 Trojans) was used. Each tank (naïve and Trojan) was fed the same diet (control and EOs diets). The cohabitation trial lasted 39 days (104 days from the beginning of the nutritional trial), considering that a minimum of 3–5 weeks is needed to successfully parasitize naïve fish under a cohabitation challenge model^130^. At the end of the challenge, naïve fish from each of the tanks (n = 15) were randomly weighed, sacrificed with an overdose of anesthetic and frozen until parasite counting. The presence of parasites was checked in all branchial arches (right and left), counted one by one in each gill filament using a stereomicroscope, and classified as adults, juveniles, post-larvae and eggs. The classification was attributed depending on the size and the number of clamps: post-larvae (small size—around 200 microns; 4–5 pairs of clamps), juveniles (medium size—around 2000 microns; 20–30 pairs of clamps) and adults (long size—5000–6000 microns; around 50–60 pairs of clamps). Prevalence, intensity and abundance were calculated according to Rózsa et al.^131^.

Animal experimental procedures were conducted in compliance with the research protocol approved by the IRTA’s Committee of Ethics and Animal Experimentation and in accordance with the Guidelines of the European Union Council (86/609/EU) for the use of laboratory animals.

### Statistics

Differences between BW were analysed with an unpaired t-test and each time point was analysed individually assuming data homoscedasticity (GraphPad PRISM 7.00). Microarrays extracted raw data were analysed with Genespring version 14.5 GX (Agilent Technologies). The 75% percentile normalization was used to standardize arrays for comparisons and data were filtered by expression. An unpaired t-test was conducted without correction to identify those DEGs between both diets. Principal component analysis (PCA), Venn diagram, and the hierarchical heatmap were obtained with Genespring version 14.5 GX. Mucous cell density and size were compared with a t-test analysis (SPSS, version 2.4). Differences in parasite number were compared by means of a Two-way ANOVA considering diets and parasite stages as independent factors. Statistical differences were set at P value < 0.05.

## Supplementary information

Supplementary Information 1.

Supplementary Information 2.

Supplementary Tables.
