# Supplementary material for: Unveiling the effect of dietary essential oils supplementation in Sparus aurata gills and its efficiency against the infestation by Sparicotyle chrysophrii
Source: Sci Rep. 2020 Oct 20;10:17764. doi: 10.1038/s41598-020-74625-5 (PMC7576129; doi:10.1038/s41598-020-74625-5)

**Supplementary file 1**

**Unveiling the effect of dietary essential oils supplementation in *Sparus aurata* gills and its efficiency against the infestation by *Sparicotyle chrysophrii***

Joana P. Firmino, Eva Vallejos-Vidal, Carmen Sarasquete, Juan B. Ortiz-Delgado, Joan Carles Balasch, Lluis Tort, Alicia Estévez, Felipe E. Reyes-López, Enric Gisbert

**Supplementary file 1.** Histochemical properties of the mucous cells and the epithelium in gills from gilthead sea bream fed experimental diets supplemented with a blend of garlic, carvacrol and thymol essential oils (DIET 1) or garlic essential oil alone (DIET 2);


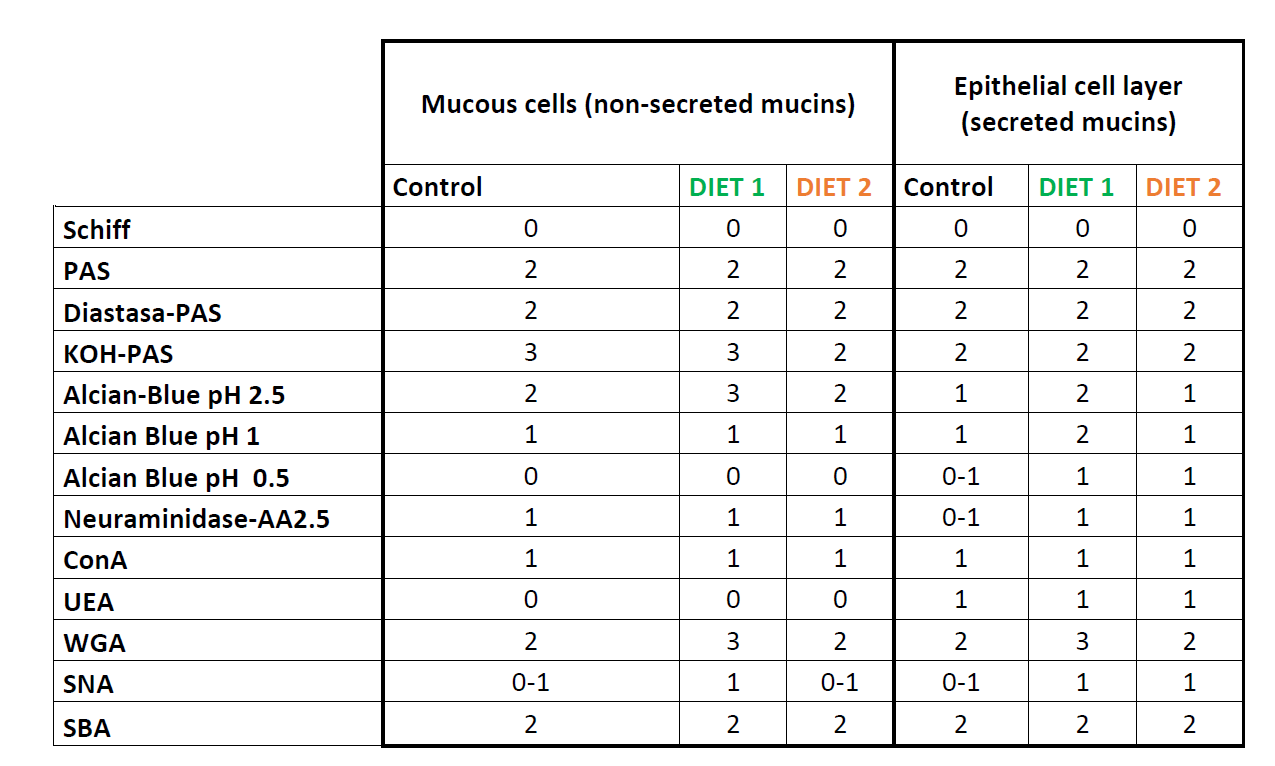


Results are expressed as the semiquantitative assessment of colour intensities by the scores of four independent observers: (0) negative; (1) weak; (2) moderate; (3) intense; and (4) very intense (see Suplementary File 1). DIET 1: Experimental diet containing a microencapsulated additive at 0.5% composed of a blend of garlic, carvacrol and thymol synthetic EOs; DIET 2: Experimental diet containing microencapsulated garlic synthetic EO at 0.5%.

Abundance of *S. chrysophrii* parasites in fish (*S. aurata*) fed with the control diet and the diet supplemented with garlic essential oil exclusively. Different ectoparasite developmental stages are represented according to their morphological characteristics: eggs, post-larvae (early juveniles with 2-4 pairs of clamps), juveniles and adults. The total load of the ectoparasite (TOTAL) is also indicated. Circles and rhombus represent parasite counting per individual fish (N = 15); mean ± standard deviation are represented. Circles (dark pink): gilthead seabream fed with control diet; Rhombus (dark yellow): gilthead seabream fed with garlic essential oil supplemented diet. *, indicate significant differences between dietary groups with corresponding adjusted P-values (*P* < 0.05).


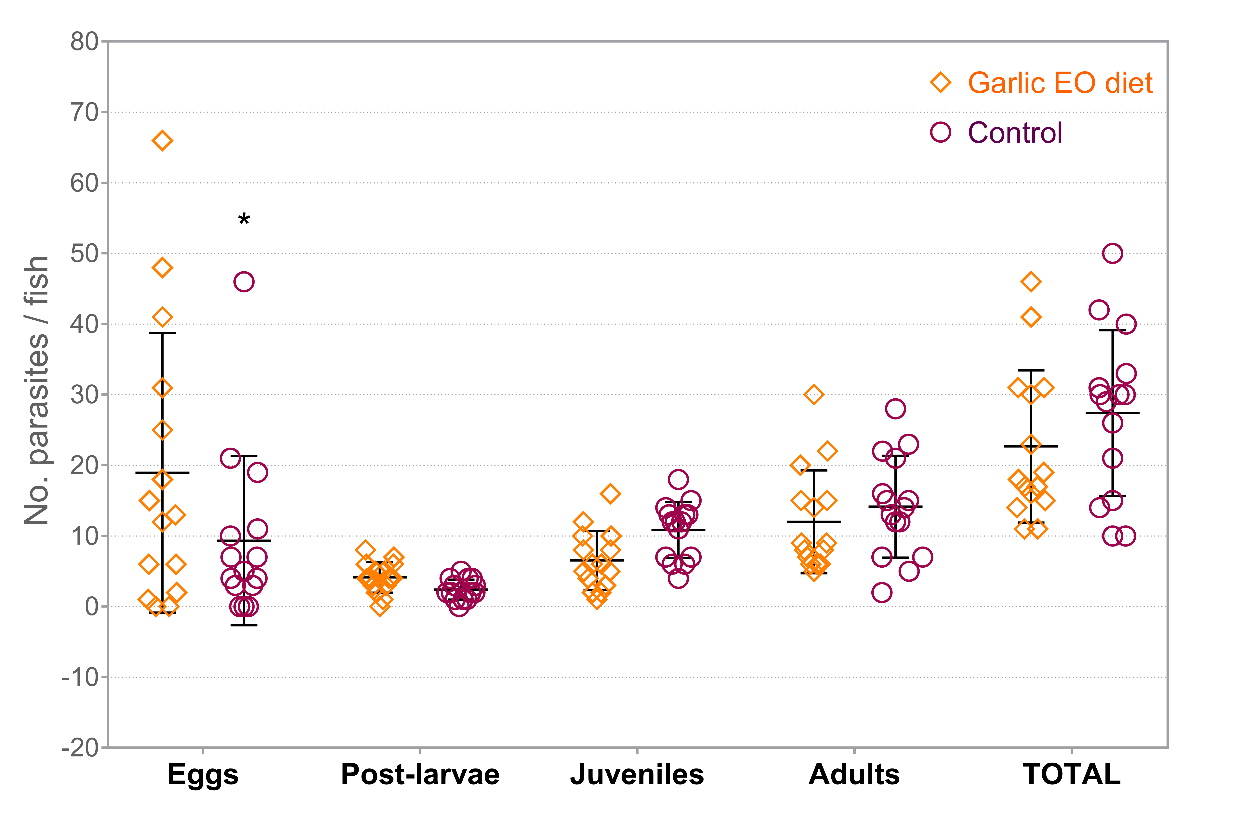

Supplement: Supplementary file 1 — Supplementary Information 1. [file 41598_2020_74625_MOESM1_ESM.docx]
