# Supplementary material for: Unveiling the effect of dietary essential oils supplementation in Sparus aurata gills and its efficiency against the infestation by Sparicotyle chrysophrii
Source: Sci Rep. 2020 Oct 20;10:17764. doi: 10.1038/s41598-020-74625-5 (PMC7576129; doi:10.1038/s41598-020-74625-5)
Supplement: Supplementary file 2 — Supplementary Information 2. [file 41598_2020_74625_MOESM2_ESM.docx]

**Supplementary file 2**

**Unveiling the effect of dietary essential oils supplementation in *Sparus aurata* gills and its efficiency against the infestation by *Sparicotyle chrysophrii***

Joana P. Firmino, Eva Vallejos-Vidal, Carmen Sarasquete, Juan B. Ortiz-Delgado, Joan Carles Balasch, Lluis Tort, Alicia Estévez, Felipe E. Reyes-López, Enric Gisbert

**Supplementary file 2.** Semiquantitative assesment of staining intensity scores used in the histochemical analysis of gills in *Sparus aurata*: (0), negative; (1), weak; (2), moderate; (3), intense; (4), very intense.
